# Ovarian cancer recurrence: is the definition of platinum resistance modified by PARPi and other intervening treatments? The evolving landscape in the management of platinum-resistant ovarian cancer

**DOI:** 10.20517/cdr.2022.13

**Published:** 2022-05-12

**Authors:** Michael J. Flynn, Jonathan A. Ledermann

**Affiliations:** ^1^Department of Oncology, University College London Hospital, London NW1 2PG, United Kingdom.; ^2^UCL Cancer Institute, University College London, London WC1E 6DD, United Kingdom.

**Keywords:** Platinum resistance, PARP inhibitors, VEGF inhibitors, immune checkpoint inhibitors, DNA damage response

## Abstract

Definitions of platinum resistance have been questioned and changed over the last five years, even though no predictive biomarker of resistance exists. These have sculpted how we approach platinum retreatment and, consequently, how we devise new treatment strategies for those patients with tumour progression on platinum therapy. Platinum-non-eligible ovarian cancer is treated with single-agent non-platinum drugs. When bevacizumab can be added to chemotherapy, progression-free survival improves significantly. For patients with a BRCA mutation, PARP inhibitor monotherapy is an option compared to chemotherapy. There is currently no clearly identified role for immune-checkpoint inhibition in this patient population. This review describes some of the challenges in treating patients with platinum resistance and suggests refinements in the selection of patients most likely to benefit from targeting a DNA damage response, angiogenesis or immune modulation. It also describes novel agents of interest and possible mechanisms of the synergy of therapeutic combinations.

## INTRODUCTION

### Background of platinum retreatment

The idea of platinum rechallenge was introduced in the 1980s at a moment in history when few treatments were available for recurrent ovarian cancer. Across several Phase II studies, the treatment-free interval (TFI) was one of the most important variables predicting response to second-line chemotherapy^[1]^. Later, Markman and Hoskins proposed that trials of new agents be stratified into primary platinum-resistant, secondary platinum-resistant, potentially platinum-sensitive and those with indeterminate sensitivity^[2]^. These definitions underwent further refinement, with variation in the cut-offs of TFI between 4 and 12 months for intermediate platinum-sensitive disease and ultimately 6 months for being considered as platinum-sensitive, with this latter definition being used for the next 3 decades^[3]^. It was first rigorously questioned at the 2010 Gynecologic Cancer InterGroup (GCIG) Ovarian Cancer Consensus meeting, during which its use was criticized as the response to platinum gradually increases with TFIp (TFI after platinum) in a non-linear way^[3]^. During the fifth GCIG consensus meeting in 2015, the terminology: platinum-sensitive and platinum-resistant in clinical trials was replaced with TFIp considered as a continuous variable among others discussed below.

### Current definitions of platinum resistance and clinicopathological predictors of platinum responsiveness

According to ESMO-ESGO consensus meeting guidelines for the management of recurrent ovarian cancer^[4]^, platinum-non-eligible ovarian cancer (PNEOC) patients are those who progress on or immediately after their last platinum-based chemotherapy or have contraindications to platinum. Platinum-eligible ovarian cancer (PEOC) includes all other cases of relapse. This includes patients without evaluable or no residual disease after primary surgery or who have relapsed following stage I disease.

There is no biomarker of platinum resistance. However, research is ongoing to define predictive biomarkers of resistance as well as prognostic markers that may be used as tools to guide treatment selection in patients with PNEOC.

For example, Lee *et al.*^[5]^ have developed a nomogram to refine prognostication in this group using six pre-treatment variables [TFIp, performance status, size of the largest tumour, cancer antigen-125 (CA-125), haemoglobin and the number of metastatic organ site]. This nomogram improved overall survival prediction in patients with PEOC compared to models with fewer prognostic factors or TFIp alone. This could have applications for stratification in clinical trials and counselling patients.

An important predictive variable of response to platinum is tumour biology and histology; for example, response rates are lower in patients with clear cell, low-grade serous and mucinous ovarian cancers^[6]^. Tumour molecular changes, including the presence of homologous recombination deficiency, increase the likelihood of a response to platinum^[7]^.

### Mechanisms of platinum resistance

#### DNA damage response detection and repair

**Figure 1:**
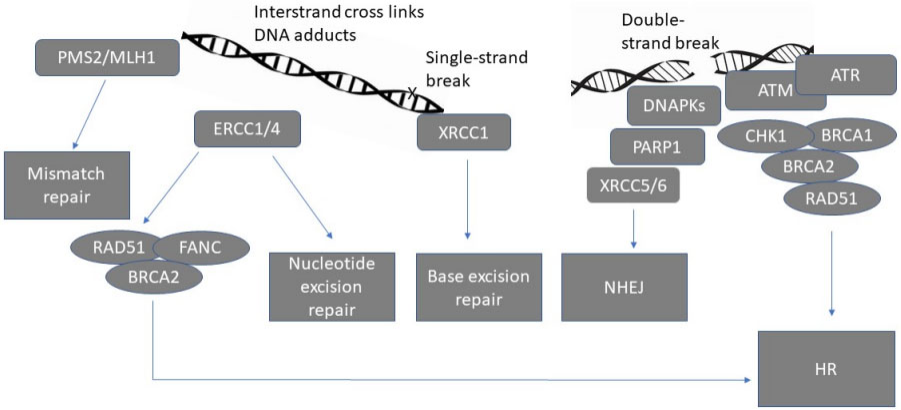
Schematic representation of major DNA repair pathways^[8]^. PMS2: postmeiotic segregation increased 2, MLH1: mutL homolog 1, ERCC1/4: Excision repair cross-complementing 1/4, RAD51: RAD51 recombinase, FANC: Fanconi anaemia complementation group, BRCA1/2 : Breast cancer gene 1/2, DNAPKs: DNA protein kinases, PARP1: Poly (ADP-ribose) polymerase, XRCC5/6: X-Ray Repair Cross Complementing 5/6, ATM: ATM Serine/Threonine Kinase, ATR: Ataxia telangiectasia and Rad3-related, CHK1: checkpoint kinase 1, HR: homologous recombination, NHEJ: non-homologous end joining.

The DNA damage response is utilised to detect DNA damage and initiate DNA repair in order to maintain genomic integrity^[8]^. It consists of a network of interrelated signalling pathways, which can be broadly divided into homologous recombination (HR) dependent and HR independent repair pathways [Figure 1].

HR dependent repair is designed to repair double-strand DNA breaks and interstrand crosslinks and, to a lesser extent, other kinds of DNA damage^[9]^. DNA repair, which is not dependent on HR, includes non-homologous end joining (NHEJ) for interstrand, double-strand breaks and intrastrand breaks. Other HR independent pathways that are less error-prone include mismatch repair, base excision repair and nucleotide excision repair (NER). These are typically recruited for the repair of single-strand breaks or damage induced by DNA adducts^[10]^.

The ERCC1-XPF nuclease enzyme is translated from the mRNAs of the ERCC1 and ERCC4 genes^[11]^. This enzyme plays a key role in DNA damage repair chiefly via NER and also that caused by interstrand crosslinks and double-strand breaks by HR and NHEJ^[11]^. ERCC1-XPF targeting is a strategy being explored to increase the sensitivity of cancer cells to some DNA-damaging chemotherapeutic agents.

The master sensors (ATM, ATR, and DNA-PKs) are large kinases that sense DNA damage and initiate repair signalling cascades by phosphorylating key proteins, which are primarily involved in HR dependent repairs such as BRCA1, CHK1, CHK2, and RAD51^[12]^. The activation of signalling transduction pathways, including PI3K/AKT, promotes the activation of DNA damage response (DDR) cell checkpoints that halt cell cycle progression, allowing more time for DNA repair^[8]^.

#### Correlation between platinum sensitivity and PARPi (PARP inhibitor) sensitivity

BRCA-deficient ovarian cancers have increased platinum sensitivity^[13-14]^. *In vitro,* reversion of BRCA mutations confers platinum and PARPi resistance^[15-16]^. In clinical studies, response to olaparib correlates with TFIp^[17]^. There is also a correlation between deficiency in other HR genes, *ex vivo* PARPi sensitivity, and platinum sensitivity in patients^[18]^. Multiple resistance mechanisms to platinum and PARPi have been described independently, although, following on from the above, significant mechanistic crossover exists.

#### Mechanisms of platinum and PARPi resistance

HR-dependent mechanisms of resistance include restoration of BRCA function by secondary or reversion mutations, or restoration of HR by loss of 53BP1, RIF1 or the shieldin complex amongst others [Table 1]. One major limitation of standard HR assays is that they are mostly insensitive to the detection of homologous recombination deficiency (HRD) reversion^[16]^. HR functional assays require viable cancer cells to be exposed to DNA damaging agents *ex vivo*, which therefore limits the access to samples and assay reproducibility.

**Table 1:** Mechanisms of PARP/platinum resistance

**Mechanism**	**Proteins involved**
PARP activity alteration loss of PARG increased stabilisation of replication forks	RAS PI3K/AKT PARP
Altered ion channel drug accumulation upregulation of drug efflux pumps Intracellular drug inactivation	VRAC MRP 2
Restoration of BRCA function through secondary reversion mutation modification of other HR proteins	BRCA1/2 53BP1 RIF1 shieldin complex

RAS = Rat sarcoma, PI3K phosphor-inositol 3-kinase; AKT: AK strain transforming; PARP: Poly (ADP-ribose) polymerase; PARG: Poly ADP (adenosine diphosphate) Ribose Glycohydrolase; VRAC: volume-regulated anion channel; MRP2: multidrug resistance-associated protein 2; BRCA1/2: Breast cancer gene 1/2; 53BP1: Tumour Protein P53 Binding Protein 1; RIF1: Replication Timing Regulatory Factor 1.

Mechanisms independent of HR include increased stabilisation of replication forks, upregulation of drug efflux pumps, PARP activity alteration, loss of PARG and RAS/PI3K/AKT pathway activation. Associated overexpression of STAT5B and RELA, two transcription factors associated with platinum resistance, is less well understood^[19]^.

Platinum resistance may also emerge due to reduced intracellular drug accumulation, for example, through reduced intracellular drug uptake, intracellular drug inactivation, enhanced DNA repair or altered apoptotic signalling pathways^[20]^.

#### Refining biomarkers of resistance to platinum and PARPi

HRD is a useful biomarker for predicting the initial response to both platinum chemotherapy and PARPi, though biomarkers of resistance require much refinement.

Standard tests for HRD, including the Myriad genomic instability score and Foundation Medicine loss of heterozygosity test, predict the presence of HRD based on genomic features^[21]^. These and other genomic tests vary in terms of the genomic features measured and the threshold definitions for identifying patients considered to have HRD. Clinically, HRD test results and PARPi responses can be discordant. This may be because tumours with reversion mutations that restore HR function still exhibit evidence of HRD on these assays or that alternative HR independent PARPi resistance mechanisms may be playing a predominant role. Functional assays of HR genes may overcome some of these challenges in predicting the presence of HRD^[21]^. The measurement of somatic mutations, such as a BRCA reversion mutation in ctDNA, is non-invasive and warrants further development^[22]^.

Approximately 40% of high-grade serous HR proficient ovarian tumours, demonstrate increased Cyclin E expression by CCNE1 gene amplification, increased copy numbers or enhanced protein expression^[23]^. These CCNE1 high tumours are associated with platinum resistance and poor survival^[24]^.

### Other tumour factors contributing to treatment resistance

#### Tumour microenvironment

Together with genomic alterations in the DNA damage response, the tumour microenvironment is an increasingly recognised contributor to our understanding of resistance mechanisms in ovarian cancer^[19]^.

The increased infiltration of immunosuppressive regulatory T cells has been correlated with enhanced tumour growth^[25]^, whereas the presence of CD8+ tumour infiltrating lymphocytes is correlated with enhanced survival^[26]^. The most predominant immune cells associated with ovarian cancer are macrophages. Tumour-associated macrophages (TAMs) are easily polarised by tumour-cell-producing colony-stimulating factor-1 into an immunosuppressive M2-like phenotype^[27]^. The main pro-tumoural function of M2-like TAMs is the secretion of cytokines and exosomes that induce microRNAs, which directly promote the survival, invasion potential and chemoresistance of ovarian cancer cells^[27]^. PD-1, PD-L1 expression and Tumour Mutational Burden have not shown consistent validity as predictive biomarkers for immune checkpoint inhibition in ovarian cancer^[19]^. Retinoic acid-inducible gene-I overexpression is correlated with platinum-resistant ovarian and other refractory cancers^[28]^. Its overexpression is associated with local immunosuppressive changes and a distinct immune signature. Extensive stromal desmoplasia has also been associated with platinum resistance^[29]^.

#### Altered metabolism in cancer tissues

Accumulating evidence suggests that tumour metabolism differs from that of matched normal tissues^[30]^, and metabolic reprogramming may cause therapy resistance. Of relevance to platinum resistance, in one cisplatin-resistant PDX ovarian cancer model glycolysis, the tricarboxylic acid and urea cycle pathways were deregulated with higher mitochondrial respiration. This may suggest a role for therapies that modulate metabolism, such as metformin. Other drugs used in non-cancer indications and new small molecule inhibitors of mitochondrial complexes are being increasingly utilised to target cancer metabolism^[31]^.

## CURRENT APPROACHES TO THE MANAGEMENT OF PNEOC

### Chemotherapy

Highlighting the clinical relevance of the arbitrariness of TFIp to decide on subsequent platinum retreatment, Lindemann* et al*.^[32]^ compared second-line platinum *vs.* non-platinum regimens in a cohort of patients who would have traditionally been regarded as platinum-resistant, i.e., those with a TFIp < 6 mo. They found a greater CA-125 response rate of 51 *vs.* 21% (*P* < 0.001) in those treated with a platinum-based therapy compared to a non-platinum regimen; and in those patients with TFIp between 3 and 6 months, improved overall survival.

Using the new and modified definition of resistance, patients with PNEOC, i.e., those progressing on platinum, are typically offered non-platinum-based chemotherapy such as weekly paclitaxel, pegylated liposomal doxorubicin (PLD) or topotecan with or without bevacizumab^[3]^. There have been comparatively few randomised phase III trials in this setting. Table 2 summarises the key data. In the CORAIL trial, comparing lurbinectedin to PLD or topotecan in patients with a TFI < 6 mo, the PFS was similar across all groups^[33]^. In the AURELIA trial, patients who relapsed after 1-2 prior lines of platinum were randomised between topotecan, PLD, or weekly paclitaxel with or without bevacizumab^[34]^. The combination of bevacizumab with PLD, weekly paclitaxel or topotecan improved mPFS compared to chemotherapy alone. Alternative non-platinum options include oral etoposide, tamoxifen, gemcitabine and treosulfan^[3]^.

**Table 2:** Phase III trials in PNEOC

**Trial**	**Treatment Arms**	**mPFS**	**Reference**
CORAIL	Lurbinectedin *vs.* control arm (PLD *vs.* topotecan)	3.5 *vs.* 3.6 mo HR = 1.057 *P* = 0.6294	Gaillard *et al. (2018)*^[33]^
ARIEL4	Rucaparib *vs. *weekly paclitaxel (TFIp 1-6 months)	6.4 *vs.* 5.7 mo HR = 0.78 (95%CI: 0.54-1.13)	* Oza (2021)* ^[36]^
AURELIA	Bevacizumab plus chemotherapy (PLD or topotecan or weekly paclitaxel) *vs. *chemotherapy alone	6.7 *vs.* 3.4 mo HR = 0.48 *P* < 0.001	Pujade-Lauraine *et al. (2014)*^[34]^
JAVELIN Ovarian 200	Avelumab plus PLD *vs. *PLD *vs. A*velumab	3.7 *vs.* 3.5 *vs.* 1.9 mo HR (combination *vs.* PLD) = 0.78 one-sided *P *= 0.03 HR (avelumab *vs.* PLD) = 1.68 one sided *P *=0.99	Pujade-Lauraine *et al. (2021)*^[41]^

PLD: pegylated liposomal doxorubicin; mPFS: median progression-free survival; mo: months; HR: hazard ratio; TFIp: platinum-free interval; CI: confidence interval.

### PARP-inhibitors

There is a role for single-agent PARP inhibitors, particularly in those with BRCA mutations that have become resistant to platinum. This is demonstrated in the single-arm Phase II QUADRA trial, in which patients were treated with niraparib after more than three lines of therapy that did not include a previous PARPi. The clinical benefit rates in patients with a BRCA1 or two mutations and TFIp < 6 months were 38% and 33%, and in the group that was platinum-refractory, 50% and 31%, at 16 and 24 weeks, respectively^[35]^. Although this is a single-arm Phase 2 study, the data does suggest an important role for niraparib in PNEOC patients with a BRCAm. ARIEL 4 (NCT02855944) is a Phase 3 study evaluating rucaparib *vs.* standard of care chemotherapy in patients with BRCA-mutated, relapsed ovarian cancer. Approximately half of the patients included in the trial had a TFIp of between 1 and 6 months, and the mPFS in this group was 6.4 months for rucaparib and 5.7 months for chemotherapy (HR 0.78, 95% CI 0.54-1.13)^[36]^.

### VEGF inhibitors

Angiogenesis is a hallmark of cancer^[30]^, with neo-angiogenesis abundantly present in ovarian cancer. Antiangiogenic therapy plus chemotherapy has shown an improvement in responses and PFS in PEOC compared to chemotherapy alone^[3]^, and improvements in PFS have also been demonstrated in patients with a TFIp < 6 months in the Phase III AURELIA trial using the VEGF-A monoclonal antibody, bevacizumab, plus chemotherapy^[34]^, or in smaller randomised Phase II trials of VEGF-R small molecule inhibitors, pazopanib with weekly paclitaxel (MITO-11)^[37]^ or sorafenib with topotecan (TRIAS)^[38]^.

In AURELIA, the combination of bevacizumab with PLD, weekly paclitaxel or topotecan improved mPFS compared to chemotherapy alone in patients who relapsed after 1-2 prior lines of platinum^[34]^ [Table 2]. However, it remains unclear based on these data when it might be appropriate to stop chemotherapy in those continuing to respond to the combination.

### Immune checkpoint inhibitors

The results of trials of Immune checkpoint inhibitors** (**ICPI) monotherapy in ovarian cancer have been disappointing. In two Phase II trials of programmed cell death protein-1/ligand-1 (PD-1/PD-L1) inhibitors, pembrolizumab and avelumab showed little benefit in ovarian cancer cohorts^[39-40]^; however, it was hoped that in subgroups of patients including PNEOC patients, they may have a niche role.

Avelumab, either alone or in combination with PLD in platinum-resistant ovarian cancer (JAVELIN Ovarian 200), failed to show a significant OS benefit compared to PLD alone [Table 2]. However, exploratory analyses suggest there may have been a benefit of the combination in those with an initial response to earlier lines of chemotherapy^[41]^. As a role for bevacizumab has been demonstrated in AURELIA, the question of whether ICPI enhances this benefit is relevant. NRG-Gy009 study (NCT02839707), which has completed recruitment, compared the combinations of PLD and bevacizumab *vs.* PLD and atezolizumab *vs.* PLD and atezolizumab and bevacizumab; the results are awaited [Table 3].

**Table 3:** Combination trials of interest

***clinicaltrials.gov*** ** identifier**	**Treatment arms**	**Proposed mechanism of synergy**
NCT02502266 (NRG-Gy005)	Olaparib *vs.* cediranib *vs*. olaparib-cediranib *vs.* investigator’s choice of chemotherapy (paclitaxel/topotecan/PLD).	antiangiogenic therapy induces a hypoxic tumour microenvironment, thereby enhancing synthetic lethality by downregulation of HR genes
NCT02839707 (NRG-Gy009)	PLD and bevacizumab *vs.* PLD and atezolizumab *vs.* PLD and atezolizumab and bevacizumab	VEGF targeting reduces inhibition of tumour immune cell suppression which permits increased efficacy of PD-L1 inhibition and chemotherapy cytotoxicity
NCT04065269 (ATARI)	ceralasertib and olaparib *vs.* ceralasertib	ATR plus PARP inhibition overcomes PARPi resistance by inducing increases in replication fork stalling, double-strand breaks, and apoptosis

PLD: pegylated liposomal doxorubicin; HR: homologous recombination; ATR: Ataxia telangiectasia and Rad3-related; PARP: Poly (ADP-ribose) polymerase; PARPi: PARP inhibitor.

Combining two ICPIs, such as anti-PD-1 and anti-cytotoxic T lymphocyte-associated-4 antibodies, may increase the activity of immunotherapy with evidence that nivolumab and ipilimumab showed a longer PFS than nivolumab alone (mPFS of 3.9 *vs.* 2 months), albeit with greater toxicity^[42]^. However, these figures are notably comparable to those seen for single-agent non-platinum-based chemotherapies.

Other trials are exploring the use of maintenance immunotherapy after chemotherapy to improve PFS. One such study is the PROMPT Phase II trial, in which patients receive pembrolizumab after 4-6 cycles of weekly paclitaxel (NCT03430700).

## NEWER STRATEGIES FOR OVERCOMING PLATINUM RESISTANCE

The above data show that with current regimens, mPFS is short, and tools to select patients likely to benefit most are required.

### Refinements in patient selection for bevacizumab 

There are currently no predictive biomarkers for bevacizumab response available in the clinic. Angiogenic markers, including micro-vessel density, CD31 expression and tumour VEGF-A levels, may provide prognostic information in recurrent ovarian cancer. These were identified in the Gynecologic Oncology Group (GOG) 218 study as potential predictive biomarkers for the use of bevacizumab^[43]^. Another retrospective analytical study showed that a signature comprising alpha-1 acid glycoprotein, mesothelin, FLT4 and CA-125 identified those patients more likely to benefit from bevacizumab^[44]^. In a concordance exploratory study of ICON7 samples, plasma concentrations of several angiogenesis-associated factors were determined using multiplex ELISAs, with high Ang1 and low Tie2 levels correlating best with PFS.

Tie1 and 2 are receptor tyrosine kinases that function as key regulators of blood vessel development and pathological processes including angiogenesis^[45]^. One observational biomarker study (VALTIVE) is currently recruiting to determine the clinical value of measuring plasma Tie2 concentrations in ovarian cancer patients who are receiving bevacizumab (NCT04523116).

### Novel treatments

#### Targeting Ataxia telangiectasia and Rad3-related

Targeting Ataxia telangiectasia and Rad3-related (ATR) is an important kinase regulating the DDR. It is responsible for sensing replication stress and signalling to cell cycle checkpoints to initiate repair^[46]^. ATR inhibitors have been shown to reduce the rate of DNA repair in cells, thereby increasing DNA damage and apoptosis^[47]^. Single-agent ATR inhibition appears to show some efficacy in PNEOC^[46]^.

#### G-Quadruplex (G4) stabilisation

G4 structures can form at thousands of sequences in the human genome and increase the propensity for DNA damage by impeding DNA polymerase, and thereby DNA damage repair processes^[48]^. CX-5461 is a small molecule RNA polymerase transcription inhibitor that selectively kills HR deficient cancer cells by stabilising G4 structures^[49]^. Phase 1 studies of CX-5461 are being investigated in solid tumours, including in a platinum/PARPi resistant ovarian cancer cohort (NCT04890613).

#### Cell cycle checkpoint inhibition

The cell cycle checkpoint regulators CHK1 and CHK2 halt cell division to allow DNA damage to be repaired before DNA replication^[50]^. Cell cycle checkpoint inhibition may thereby prevent the progression of cancer cells through the cell cycle, halting replication and tumour progression. WEE-1 inhibitors block the activity of WEE-1 kinase, a G2 cell-cycle checkpoint, and enhance cancer cell apoptosis^[51]^.

Prexasertib is one example of a CHK1 inhibitor, which demonstrated responses in a phase II trial that were most marked in patients with platinum-resistant ovarian cancer^[52]^. A phase II study of the combination of AZD1775, a WEE-1 inhibitor and carboplatin in platinum-resistant ovarian cancer, demonstrated an ORR of 43%^[53]^.

#### Epigenetic re-sensitisation

Treatment resistance is often associated with the accumulation of epigenetic changes^[54]^. It has therefore been hypothesised that epigenetic modulation may re-sensitise tumours to platinum chemotherapy.

The DNA methyltransferase (DNMT) and Histone Deacetylase inhibitors have shown little activity as single agents in platinum-resistant ovarian cancer. However, in combination, they may enhance sensitivity to platinum by altering epigenetic regulation of gene expression. In a randomised phase II study assessing the DNMT inhibitor, guadecitabine in combination with carboplatin *vs.* investigator’s choice of chemotherapy, the PFS rate at 6 months was 37% *vs.* 11%^[55]^. The DNA damage initiated by DNMT inhibitors is repaired by the BER pathway, in which PARP1 plays a key role, and therefore there may also be a rationale to combine DNMT and PARP inhibition.

### Combination approaches

#### Angiogenesis and PARPi

Antiangiogenic therapy has been shown to induce a hypoxic tumour microenvironment associated with the downregulation of HR genes^[56]^, providing the rationale to enhance the synthetic lethality of PARPi with angiogenesis inhibitors which also separately work to interfere with angiogenesis.

The Phase II AVANOVA2 trial compared the combination of niraparib and bevacizumab *vs.* single-agent niraparib in patients with PEOC^[57]^. Niraparib plus bevacizumab significantly improved mPFS compared with niraparib alone (11.9 mo *vs. *5.5 mo; HR 0.35, *P* < 0.0001) and has provided a rationale to test this strategy in PNEOC.

EVOLVE was a phase II trial of cediranib-olaparib in ovarian cancer progressing on PARPi, recruiting a cohort of patients who were also defined as platinum-resistant, with 2/10 patients in this cohort demonstrating a PR^[58]^. The anti-tumour activity of this combination continues to be assessed in the randomised Phase III NRG-Gy005 (NCT02502266) trial currently recruiting patients with platinum-resistant ovarian cancer to receive either olaparib, cediranib, olaparib-cediranib or investigator’s choice of chemotherapy (paclitaxel/topotecan/PLD) [Table 3].

#### DDR response

In a large panel of acquired and *de novo* PARPi- and platinum-resistant CCNE1 amplified *in vitro* and PDX models, ATR and PARPi synergy was demonstrated^[59]^. This, amongst other data, has provided the rationale for the combination of ceralasertib and olaparib for recurrent platinum-resistant ovarian cancer in CAPRI^[60]^. Although no objective responses were demonstrated, the combination was well tolerated, and in two patients with BRCA1 mutations, a 50% fall in CA-125 was seen.

It will be interesting to see the data from the combination arm of ATARI (NCT04065269) in platinum-resistant ovarian clear cell cancer and carcinosarcomas, which may provide insights into how the combination of these drugs may alter the DDR in those subgroups of patients that do not classically display responsiveness to chemotherapy and PARPi [Table 3].

There is a clear rationale to combine other drugs regulating the DDR described earlier in this review, for example, WEE1 inhibitors, with platinum-based chemotherapy and PARPi.

#### Immunotherapeutic combinations

An alternative strategy is the combination of an ICPI with a PARPi, chemotherapy or other DDR modifying drugs. One possible mechanism of synergy is the observation that PARPi can activate the STING (stimulator of interferon genes) pathway to increase T-cell tumour infiltration^[61]^. TOPACIO was a Phase 1/2 trial testing niraparib plus pembrolizumab in platinum-resistant ovarian and triple-negative breast cancer patients. A subgroup analysis of the ovarian cancer cohort showed that the combination was promising for patients without HR deficiency^[62]^. There was a small cohort of patients in this group, and therefore other larger studies will need to focus on immunogenomic profiling to select patients most likely to benefit from this strategy.

## CONCLUSION

Although the definition of true resistance to platinum-based chemotherapy has changed over the last four decades, few treatments have significantly changed outcomes in the vast majority of patients in this cohort. Next-generation sequencing has become faster and more affordable due to automation, which is permitting standardisation of techniques for analysing liquid biopsies and immunogenomic profiling. These refinements may lead to an improvement in patient selection for some of the novel strategies and combinations discussed in this review. Biomarker-driven trial designs will accelerate the better selection of and sequencing of treatment lines, including those targeting immune modulation, modification of the DNA damage response and angiogenesis inhibition.

## DECLARATIONS

### Copyright

© The Author(s) 2022.
